# Non-Canonical Roles of Dengue Virus Non-Structural Proteins

**DOI:** 10.3390/v9030042

**Published:** 2017-03-13

**Authors:** Julianna D. Zeidler, Lorena O. Fernandes-Siqueira, Glauce M. Barbosa, Andrea T. Da Poian

**Affiliations:** Instituto de Bioquímica Médica Leopoldo de Meis, Universidade Federal do Rio de Janeiro, Av. Carlos Chagas Filho, 373, CCS, Bl. E, sala 18, Rio de Janeiro 21941-90, Brazil; julianna.zeidler@bioqmed.ufrj.br (J.D.Z.); losiqueira@bioqmed.ufrj.br (L.O.F.-S.); glaucemb@bioqmed.ufrj.br (G.M.B.)

**Keywords:** dengue virus, non-structural proteins, physiopathology, immunity, metabolism

## Abstract

The Flaviviridae family comprises a number of human pathogens, which, although sharing structural and functional features, cause diseases with very different outcomes. This can be explained by the plurality of functions exerted by the few proteins coded by viral genomes, with some of these functions shared among members of a same family, but others being unique for each virus species. These non-canonical functions probably have evolved independently and may serve as the base to the development of specific therapies for each of those diseases. Here it is discussed what is currently known about the non-canonical roles of dengue virus (DENV) non-structural proteins (NSPs), which may account for some of the effects specifically observed in DENV infection, but not in other members of the Flaviviridae family. This review explores how DENV NSPs contributes to the physiopathology of dengue, evasion from host immunity, metabolic changes, and redistribution of cellular components during infection.

## 1. Introduction

A large set of arthropod-borne diseases is caused by enveloped viruses of the Flaviviridae family, which includes dengue virus (DENV), yellow fever virus (YFV), Japanese encephalitis virus (JEV), West Nile virus (WNV), Zika virus (ZIKV), and about 70 other members. The Flaviviruses genome is a positive-sense single-strand RNA molecule of about 11 kb translated into a polyprotein that is cleaved to generate three structural (capsid, membrane, and envelope—C, prM, E) and seven non-structural proteins (NSPs—NS1, NS2A, NS2B, NS3, NS4A, NS4B, and NS5), which share functional and structural features among the different members of the family [1]. In addition to the similarities, the different outcomes of the distinct diseases caused by the flaviviruses may be partially explained by non-canonical functions of the viral proteins, which have evolved independently in each virus type. Here we considered the canonical roles those that are shared among all (or most) members of the Flaviviridae family, while the non-canonical are those exclusive to a specific virus or not generalized throughout the family. For instance, flaviviruses’ NS1 is known to interfere with the proteins of the complement system, but DENV NS1 has unique interaction partners and mediates this function in a particular manner [2,3,4,5]. Viral structural proteins, which dictate targeted-cell type specificity and the architecture of viral particles, may also have non-canonical functions, but this is beyond the scope of this review. As NSPs are dispensed from structural roles in mature virus particles, they are more prone to display multifunctional and non-canonical roles, and more recently have been largely studied probably due to their potential as targets for clinical intervention.

DENV is endemic in more than 100 countries and recent estimates predict about 390 million infections per year [6]. Taking into account the high number of dengue cases and the increased geographical extension of the disease, dengue is considered a global public health problem with deep social and economic implications. The clinical manifestations of dengue vary from a mild fever to life-threatening severe diseases (occurring in a small proportion of cases), known as dengue hemorrhagic fever (DHF) and dengue shock syndrome (DSS), which are characterized by an increase in vascular endothelium permeability leading to plasma leakage, which may evolve into a fatal hypovolemic shock. Although a number of dengue vaccines have been developed, some being in clinical trials, they have shown to be limited with regard to low immunogenicity and partial protection against different DENV serotypes [7]. Among these vaccines, Dengvaxia (developed by Sanofi Pasteur, Lyon, France) has been licensed in several countries, but it is still a matter of concern due to the reported low protection against DENV serotype 2, associated with an increased incidence of hospitalization due to severe dengue of seronegative individuals in the third year after the first dose [7,8]. While vaccination strategies are in development and improvement stages, dengue treatment is still mainly based on supportive clinical interventions that do not always prevent the evolution to the severe forms of the disease [9,10].

## 2. The Canonical Roles of Flaviviruses’ NSPs

**Figure 1:**
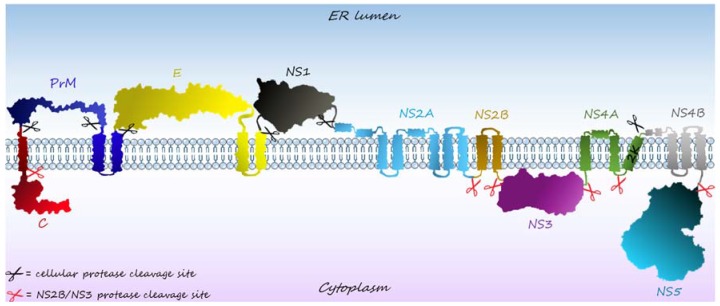
Schematic representation of the flaviviruses’ polyprotein. Viral RNA encodes a polyprotein that is co- and post-translationally processed by host proteases (black scissors) or by the viral protease NS2B/NS3 (red scissors) to generate the structural (C, PrM, and E) and non-structural proteins (NS1, NS2A, NS2B, NS3, NS4A, NS4B, and NS5), represented in different colors.

Before discussing the particular, non-canonical, roles of DENV proteins in infection and disease establishment, we will summarize, in this topic, the functions carried out by each NSP that are shared among the members of the Flaviviridae family. Most of these NSP canonical functions are related to viral replication, which depends on the assembly of a membrane-bound multi-protein replication complex (RC) [11,12,13,14], formed by the association of different NSPs with host co-factors on interconnected lipid vesicles derived from the endoplasmic reticulum (ER) [12,13,15,16]. At these sites, viral RNA is transcribed and translated into a polyprotein, which is cleaved by host and viral proteases to originate the individual viral proteins (Figure 1).

NS1 is a conserved glycosylated protein that may occur in different oligomeric forms during the virus replication cycle, namely immature monomers in the ER lumen, stable hydrophobic homodimers able to interact with membranes, or secreted soluble hexamers harboring a large central 10 nm-diameter open barrel filled with lipids [17,18,19,20]. In early stages of infection, the dimeric NS1 associates to the viral RC on the ER membrane, probably interacting with the transmembrane proteins NS4A and NS4B [21,22,23,24]. In late infection, the secreted hexamers interact with proteins of the complement system, counteracting the cellular responses to infection [4,22,23,25,26].

NS3 behaves as a protease when it uses NS2B as a co-factor, cleaving the polyprotein at specific sites and host proteins that would impair the establishment of the infection [27,28,29]. NS3 also plays a role in viral RNA replication, acting as an RNA helicase, nucleoside 5’-triphosphatase (NTPase), and RNA 5’-triphosphatase (RTPase) [24].

NS5 is the most conserved protein among flaviviruses’ NSPs, being responsible for the capping, methylation, and replication of the viral genome [30,31,32,33]. A NS5 dimer associates with NS3 and NS2B, to form the RC on ER-derived membranes. This trimeric complex is essential for the establishment of the protein-protein and protein-RNA interactions necessary for the polymerization reaction, as also observed for the polymerases of other viruses [34].

The functions carried out by the transmembrane proteins NS2A, NS2B, NS4A, and NS4B are less understood and, for this reason, it is difficult to define the canonical roles of these proteins. They do not have a known enzymatic activity, but act as scaffolds for RC formation [1,11]. For Kunjin virus (KUNV), it was shown that NS2A co-localizes with double-stranded RNA formed during genome replication, binding to the viral RNA 3’UTR, and to NS1, NS3, and NS5 to form the RC [31]. NS2A also participates in the rearrangement of cellular membranes observed during the replication of flaviviruses [32]. NS2B acts as a cofactor for NS3 proteolytic activity. NS4A and NS4B are connected by a transmembrane peptide named 2K, which is cleaved during NS4A maturation. 2K peptide maintains NS4B associated to ER, even after its cleavage from the polyprotein [33]. NS4A induces the rearranging of ER membranes seen during the infection [35]. In addition, besides participating in viral replication, NS4A promotes autophagy, preventing infection-induced cell death [36]. For WNV, it was observed that NS4A controls the ATPase activity of NS3, while for DENV, NS4B was found to interact with the NS3 helicase domain, assisting its dissociation from the RNA strand. Thus, it seems that NS4A and NS4B work co-operatively during viral replication [37].

## 3. Participation of DENV NSPs in Dengue Physiopathology

DHF/DSS pathogenesis encompasses a series of events driven by a cytokine storm triggered during infection that leads to an abrupt increase in endothelial permeability followed by plasma leakage, disseminated intravascular coagulation, and hemorrhage, which may progress to a fatal hypovolemic shock [9]. Among the possible molecular players that mediate these events, DENV NS1 is the more extensively studied. High levels of NS1 are found circulating in the blood of patients [38], and this correlates with the development of DHF [39,40]. Additionally, the detection of NS1 in patients’ sera has driven the development of diagnostic tests for dengue [39,40]. Although there are a number of questions regarding this issue that are still not completely answered, some findings start to shed light in the roles of DENV NS1 during infection and disease. 

The hexameric form of NS1 circulates associated triglycerides, cholesterols, and phospholipids, a content similar to that present in high-density lipoproteins (HDL) [41]. This, together with the fact that NS1 hexamers were shown to have tropism to the liver when injected in mice [42], and that NS1 internalization was observed in human-cultured hepatocytes, suggested that NS1 would carry lipids in the plasma of dengue patients from tissues to the liver. The implications of these findings are still not understood, but considering that lipoproteins participate in vascular homeostasis, it was hypothesized that the transport of lipids by NS1 may play a role in dengue physiopathology [41]. 

Many other studies point to a direct role of NS1 in a crucial event of severe dengue physiopathology: the plasma leakage. For instance, antibodies produced against NS1 during infection cross-react with surface antigens of endothelial cells and platelets, possibly contributing to the plasma leakage, dehydration and hypovolemic shock that are observed in the severe forms of dengue [43,44,45]. NS1 was shown to bind to both prothrombin and thrombin in dengue patients’ sera. Thrombin formation is the center of the coagulation cascade, which starts with the release of tissue factor from damaged cells and subsequent activation of an amplifying cascade generating large amounts of factor X from a few initial signaling molecules [46]. Factor X activates prothrombin, generating thrombin, which is able to cleave the fibrinogen to insoluble fibrin fibers, besides being able to cleave prothrombin itself and activating factor XIII, accelerating the coagulation reaction. In vitro assays revealed that recombinant NS1 did not interfere with thrombin activity, but it inhibited prothrombin activation and prolonged the activated partial thromboplastin time (APTT). Thus, NS1 may be responsible for abnormal APTT usually found in the first weeks of dengue onset, and possibly also contribute to plasma leakage by antibody-independent mechanisms that occur in the severe forms of the disease [47]. Additionally, NS1 was found to activate mouse macrophages and human mononuclear cells, inducing the production of proinflammatory cytokines/chemokines via activation of Toll-like receptor 4 (TLR4), which promote the disruption of endothelial cell monolayer integrity and vascular leak, leading the authors to consider NS1 a viral toxin that acts similarly to bacterial endotoxin lipopolysaccharide (LPS) [48]. Accordingly, inoculation of NS1 in mice caused endothelial dysfunction leading to plasma leakage [49]. Another mechanism proposed for NS1-mediated endothelial hyperpermeability includes the induction of sialidases and heparanases expression and activation of the lysosomal protease cathepsin L in endothelial cells, resulting in the degradation of the glycocalyx barrier, a key regulator of vascular permeability [50]. The evidences presented above suggest that NS1-induced plasma leakage is a complex phenomenon resulting from a series of events encompassing endothelial cells, cells from the immune system, and platelets.

Cellular activation and production of large amounts of immune mediators (the “cytokine storm”) are known to contribute to dengue pathogenesis and some evidence points to the contribution of DENV NSPs to this scenario. Monomeric NS1 is hydrophilic, but its dimeric form associates to membranes [17,21] and this seems to happen due to the presence of the glycosylphosphatidylinositol (GPI) anchor in NS1 [51], a post-translational modification that targets proteins to membranes [52]. Antibody-induced signal transduction is a common feature of GPI-linked proteins [53,54,55] and this was found to happen with NS1, being another possible determinant that contributes to cellular activation, cytokine storm, and dengue pathogenesis [51]. Regarding other DENV NSPs, the expression of NS5 was shown to promote the induction of IL-8 expression and secretion in HEK293-transfected cells [56], and both NS5 and NS4B induce the secretion of immunomediators in THP-1 monocytes, being this action enhanced when NS4B is linked to the 2K peptide [57]. In addition, it was observed that the conditioned media of 2K-NS4B-transfected THP1 cells contained IL-8 and TNFα in levels that induce endothelial cell permeability and increased expression of adhesion molecules [58]. NS5 also interacts with death-domain-associate protein (Daxx), a transcription repressor shown to be important to induce expression of the cytokine RANTES [59]. In addition, the ability of NF-κB to bind RANTES promoter is increased in cells expressing NS5 [60], suggesting that NS5 may contribute to DENV pathogenesis by inducing increases of cytokine expression.

## 4. Evasion from Host Innate Immune Response

**Figure 2:**
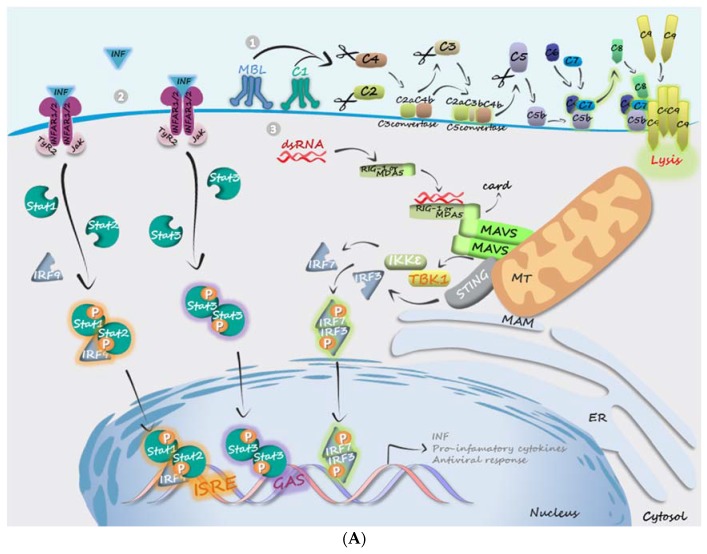

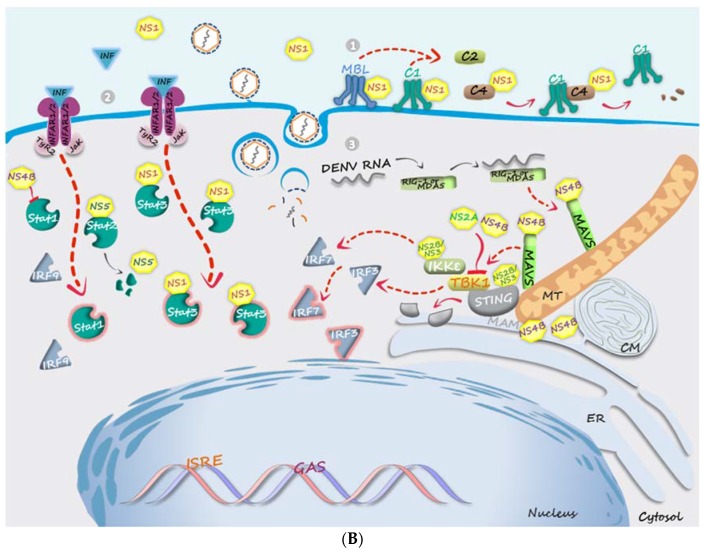
Involvement of DENV NSPs in the evasion of host innate immune response. (**A**) Pathways of immune response affected by DENV NSPs. (1) Activation of the complement system is triggered by different pathways that converge to the cleavage of factor C3 by the protease C3 convertase. This enzyme formed by the association of two other cleavage products: C4b, a fragment of C4, and C2a, a fragment of C2. The cleavage of C4 and C2 may be catalyzed by two different pathways: the classical pathway, triggered by C1 binding to antigen-antibody complexes, or by the lectin pathway, in which a carbohydrate recognition receptor, such as mannose binding lectin (MBL), associates to a serine protease after binding to carbohydrates. One of the products of C3 cleavage, C3b, binds to C3 convertase changing its substrate specificity, so that the enzyme becomes a C5 convertase. The fragment C5b, generated from the cleavage of C5, binds to the infected cell membrane, initiating the assembly of a complex formed by C6, C7, C8, and C9, which promotes cell lysis. (2) Viral dsRNAs produced during the replication of RNA viruses are recognized by PRRs. Binding of dsRNA leads the cytosolic PPRs to associate with the mitochondrial antiviral signaling protein (MAVS) through its caspase-recruitment domain (CARD), recruiting the TANK-protein kinase 1 (TBK1) and IκB kinase-ε (IKKε). These kinases phosphorylate IRF-3, which forms homodimers or heterodimers with IRF-7, which, in turn, translocate to the nucleus, inducing the expression of type I IFN and pro-inflammatory cytokines. This pathway also includes the participation of the adaptor protein STING, which acts in mitochondrial-associated membrane (MAM) to mediate RIG-I downstream signaling. (3) Type I IFN-mediated antiviral responses occurs via the expression of several IFN-stimulated genes (ISGs). IFN binding to its heterodimeric IFN-α receptor (IFNAR1/2) activates Janus kinase 1 (JAK1) or tyrosine kinase 2 (TYK2), leading to the phosphorylation of the signal transducer and activator of transcription (STAT) proteins, which dimerize and translocate to the nucleus. STAT1–STAT2 heterodimer binds to IFN regulatory factor 9 (IRF9) and migrates to the nucleus, inducing the expression of ISGs through its binding to the IFN-stimulated response elements (ISRE). Type I and type II IFNs can also induce dimerization of STAT3, which translocate to the nucleus, where it binds to gamma-activated sequences (GAS), stimulating the production of both pro- and anti-inflammatory cytokines; (**B**) Participation of DENV NSPs in the evasion of the host immune response. (1) DENV NS1 inhibits complement activation by interacting with different components of the complement system, including C1 proenzyme, C1s, C4, C4b, and MBL. The formation of the complex C4-NS1-C1s/C1 results in degradation of C4, impairing the formation of C3 convertase. NS1 binding to MBL protects DENV against MBL-mediated virus neutralization by the lectin pathway of complement activation. (2) DENV NSPs impair the innate immune response mediated by viral dsRNA recognition. NS4B interacts with the CARD domain of MAVS, impairing its binding to the cytoplasmic PRRs. Moreover, this protein, by inducing the formation of convoluted membranes (CM) and promoting mitochondrial elongation, inhibits the translocation of PRRs to MAMs. NS2B/NS3 interacts with IKKε and cleaves STING and NS2A together with NS4B, inhibiting the phosphorylation of TBK1 and its substrate IRF3. These steps impair the activation of transcription factors IRF-3 and IRF-7. (3) DENV NSPs inhibit INF-stimulated signaling in different points. NS4B interacts with STAT1, blocking its phosphorylation, and NS5 mediates STAT2 degradation, so both proteins inhibit the expression of ISGs by interfering in ISRE activation. Additionally, NS1 interacts with STAT3, inhibiting the formation of its homodimers, thus preventing GAS-induced gene expression. Red arrows represent the events induced by NSPs, while dashed red arrows represent those ones that are blocked by NSPs. ER, endoplasmic reticulum; MT, mitochondria.

To establish a successful infection, viruses need to escape from the complex and robust host immune defenses. The known mechanisms by which DENV evades the host immune system were previously reviewed elsewhere [61,62]. Here, we intend to provide an update of the more recent findings on this issue, focusing on the roles of NSPs. Many DENV NSPs interfere with the host immune response by subverting complement activation, pathways triggered by pattern recognition receptors (PRRs), as well as interferon (IFN)-mediated signaling pathways (Figure 2). It is interesting to note that each DENV NSP acts on multiple points of cellular immune response, revealing the multifunctionality of viral proteins and supporting the idea that viral apparatus evolved to constitute a robust and efficient network against an equally complex system, such as the cellular innate immune system. 

The complement system is an important component of the immune response, linking adaptive and innate immunities. Activation of the complement system is triggered by different pathways, as depicted in detail in Figure 2A. Flaviviruses’ NS1s are known to interfere with the complement system at different points [2]. In the case of DENV, NS1 inhibits both the classical and the lectin pathways (Figure 2B). NS1 N-linked glycans interact with C4, C4b, C1s proenzyme, and C1s, especially when NS1 is arranged in its hexameric form [3]. The formation of the C4-NS1-C1s/C1 complex results in C4 degradation, impairing complement activation [2]. In addition, DENV NS1 binds and recruits C4BP to the cellular membrane. C4BP is, a negative regulator of complement pathways that promotes dissociation of C4bC2a C3 convertase and acts as a cofactor for cleavage of C4b, so that NS1 binding inactivates C4b on the cell membrane [4,63]. NS1 also interacts with C1q, but the effects of this interaction on inactivation of complement cascade have not been explored so far [5]. Regarding the lectin pathway, MBL has been identified as an NS1 target both in mammalian and mosquito cells [64], protecting infected cells from immune recognition and impairing virus neutralization by complement activation. 

Innate immune response against viruses depends on the recognition of pathogen-associated molecular patterns (PAMPs) by PRRs, which trigger signaling pathways that ultimately activate the production of type I IFN and pro-inflammatory cytokines (Figure 2A). Viral RNA recognition is mediated by proteins of the DExD/H-box RNA helicases family, such as the retinoic acid-inducible gene I (RIG-I) and melanoma differentiation-associated gene 5 (MDA5) [65]. Briefly, the interaction between these receptors and the viral RNA allows it to associate to the mitochondrial antiviral signaling protein (MAVS), which activates TANK-protein kinase 1 (TBK1) and IκB kinases (IKK) [66]. These kinases mediate the activation of IFN regulatory factors (IRF), which migrate to the nucleus inducing the expression of type I IFN and pro-inflammatory cytokines. Recently, an adaptor protein named as “stimulator of the interferon gene” (STING) has also been identified as a downstream effector of RIG-I [67]. Mitochondrial-associated membranes (MAM), regions of the ER that are closely juxtaposed to mitochondria and constitute sites of communication and lipid exchange between these organelles, are important for MAVS signaling during an infection, and processes that lead to MAM disruption block the interaction between MAVS and STING, inhibiting the downstream antiviral signaling [67]. With regard to activation of PRR-mediated pathways, our group has shown that DENV infection induces the production of IFN-I and pro-inflammatory cytokines in a manner dependent on the activation of RIG-I [68]. To alleviate host antiviral response, DENV interferes with this pathway at multiple points. It was found that NS2B/NS3 interacts with IKKε, masking the kinase domain and consequently preventing the phosphorylation of IRF3 [69]. Similarly, NS2A and NS4B were shown to inhibit phosphorylation of TANK-binding kinase (TBK1) and its substrate IRF3 [70]. Another member of the DExD/H box helicases family that has a less clear role in innate immune response, DDX21, is a target of DENV NS2B/NS3 protease and its degradation was shown to facilitate DENV replication [71]. NS2B/NS3 also cleaves/inactivates STING, inhibiting the TBK1-mediated IFN expression [28,29]. DENV NS4B was shown to interact with C- and N-terminal domains of MAVS, including its CARD domains, to which RIG-I binds, probably suppressing the oligomerization of MAVS, abrogating its interaction with downstream adaptor proteins and inhibiting IFN production [72]. In addition to participating in the induction of convoluted membranes (CM) in ER (see also the next topic), recently it was shown that NS4B also induces mitochondria elongation at ER-mitochondria contact sites, which was shown to favor DENV replication by impairing translocation of RIG-I to MAMs, impairing the innate immune response [73].

Finally, DENV NSPs interfere with the IFN signaling pathway, an essential host defense against many viruses, including the flaviviruses. Type I IFN (IFNα/β) mediates antiviral responses in an autocrine and paracrine fashion by increasing the expression of hundreds of IFN-stimulated genes (ISGs) [74]. Briefly, binding of type I IFNs to the heterodimeric IFNα receptor (IFNAR, which is composed of the IFNAR1 and IFNAR2 subunits) activates Janus kinase 1 (JAK1) and tyrosine kinase 2 (TYK2), which phosphorylate the signal transducer and activator of transcription (STAT) proteins that, when phosphorylated, dimerize and translocate to nucleus (Figure 2A). STAT1–STAT2 heterodimers associate to IFN regulatory factor 9 (IRF9), and this trimeric complex binds to IFN-stimulated response elements (ISRE), activating the transcription of ISGs. Type I and type II IFNs can also induce homodimerization of STAT1 or STAT3, which translocate to the nucleus and bind to gamma-activated sequences (GAS), stimulating the production of either pro- or anti-inflammatory cytokines. DENV NS1 was identified as a ligand of human STAT3 protein [75], but the implications of this finding are still poorly understood. DENV NS4B, as well as NS4B of other flaviviruses, such as West Nile and yellow fever viruses, has anti-IFN activity [76] by blocking STAT-1 phosphorylation in cells stimulated with IFN [77]. At least for DENV, the 2K peptide is required for the anti-IFN function. In addition, the co-expression of cleaved NS4A and NS4B (mediated by NS2B/NS3 protease) contribute to further ISRE promoter inhibition [76]. So, NS4A, NS4B, and NS2B/NS3 together seem to promote an efficient inhibition of IFN-stimulated signaling pathway during DENV infection. Furthermore, a high-throughput yeast two-hybrid screening showed that flaviviruses’ NS5 (including of DENV NS5) also inhibits IFN-mediated signaling [78]. In the case of DENV NS5, it was shown that it mediates STAT2 degradation [79]. Thus, it seems that inhibition of the IFN pathway is important for flaviviruses’ infection and different DENV NSPs cooperate to perform anti-INF functions (Figure 2B).

## 5. Metabolic Alterations

Alterations in host cell metabolism caused by virus infection have been studied for many years. One of the first investigations in this sense was published in 1928 by Crabtree, where he described changes in oxidative/glycolytic patterns of tissues infected by viruses when compared to the non-infected tissues [80]. Now it is clearer that virus-induced host metabolic re-programming can affect diverse pathways, including not only glycolysis and oxidative phosphorylation, but also the pentose phosphate pathway, pyrimidine, fatty acids, and glutamine metabolisms [81]. These metabolic alterations usually favor viral replication by increasing cellular energy charge and/or supplying substrates required for production of viral progeny.

In the case of DENV, a crescent number of reports on virus-induced metabolic alterations is now appearing in the literature. For instance, our group showed that human hepatocytes infected with DENV displayed morphologically altered and uncoupled mitochondria, decreasing cellular energy charge and causing metabolic stress [82]. Other studies have shown that DENV is able to induce changes in specific metabolic routes in host cells, such as an increase in glycolysis in primary human foreskin fibroblasts [83] and an increased autophagy-mediated mobilization of fatty acids in human hepatocytes [84]. However, the molecular players that mediate these metabolic changes have only started to be elucidated.

**Figure 3:**
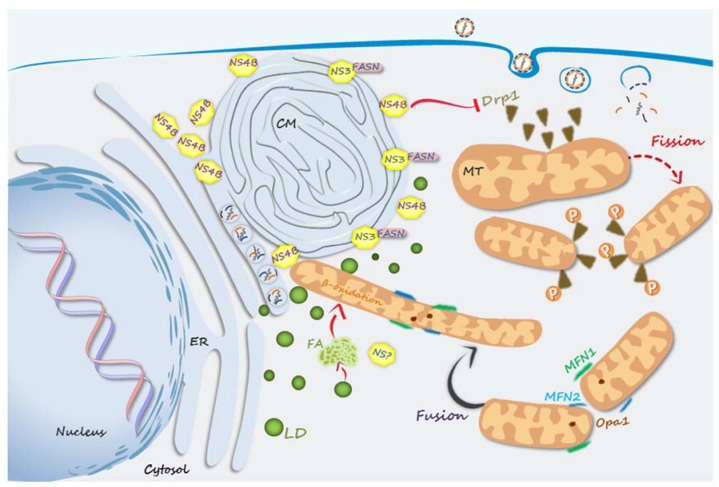
DENV NSPs-induced host metabolic alterations. During infection, DENV NS4B induces the formation of the convoluted membrane (CM), the membranous structure where viral polyprotein is processed and the resulting proteins accumulate. NS3 recruits the enzyme fatty acid synthase (FASN) to the virus replication sites, besides stimulating its enzymatic activity, increasing de novo FA biosynthesis, which provides lipids for inducing the formation of CM, as well as for the assembly of the viral envelope. Fatty acids (FA) are mobilized from lipid droplets (LD) to undergo β-oxidation mitochondria, providing energy to the high-energy demanding virus replication process. NS4B is able to inhibit phosphorylation of cytoplasmic protein dynamin-related protein 1 (Drp1), preventing the mitochondrial fission process. Fused mitochondria accumulate in infected cells, increasing the efficiency of the oxidative metabolism. Red arrows represent the events induced by NSPs, while dashed red arrows represent those ones that are blocked by NSPs. MFN1, mitofusin 1; MFN2, mitofusin 2; Opa1, optic atrophy protein 1; ER, endoplasmic reticulum; MT, mitochondria.

A typical cellular alteration observed during flaviviruses infection is the formation of virus-induced ER membrane rearrangements, which, just like a virus factory, creates a subcompartimentalization for the viral replication cycle steps—RNA translation and replication, as well as the virion assembly—allowing proper environments for the coordinated performance of each process without mutual interference. Each flavivirus induces a particular membrane rearrangement, and in the case of DENV, at least three distinct ER membrane-derived structures are formed: single membrane invaginations into the ER lumen (resembling vesicle packets), where the RC is assembled and genome replication takes place; unstructured convoluted membranes (CM), where viral polyprotein is processed and the resulting proteins accumulate to be later used for RC and virion assembly; and membranes associated with the assembly of new viral particles [85,86]. For mounting such a complex structure, which practically consists in an new organelle induced by infection, cellular metabolism should be directed to the mobilization of different substrates, especially lipids, which are essential for membrane formation, besides consisting of an important energy source for virus replication. In this context, an interesting finding was that NS3 interacts with fatty acid synthase (FASN), recruiting this enzyme to sites of viral replication [87] (Figure 3). In addition, interaction between recombinant NS3 and FASN was shown to stimulate FASN activity in vitro, suggesting that this would be a means of increasing de novo FA biosynthesis during DENV infection. The role of NS3 in redistributing FASN to sites of viral replication was later found to be dependent on NS3 interaction with the GTPase Rab18 [88], a protein localized on lipid droplets (LD) which mediate the apposition of these organelles and the ER-derived membranes, enhancing the formation of LD-associated membranes (LAM) [89]. On the other hand, in agreement with the increased requirement of energy supplies during virus replication, DENV infection was shown to increase fatty acid (FA) β-oxidation [84]. At first glace, this would seem contradictory, but it can be explained by differences in subcellular localization of each process: while FA biosynthesis occurs at viral replication sites, providing lipids for membrane synthesis, FA seems to be mobilized from LD to be oxidized in mitochondria, providing energy for virus replication. In addition to activating FA synthesis at the sites of viral replication, NS3/Rab18-induced LAM may facilitate the removal of FA from LD for β-oxidation, which, as mentioned, is increased during infection [84]. So, by managing subcellular localization of specific cellular components, DENV NSP can coordinately stimulate opposite processes that are equally important for formation of new viral particles. 

Mitochondria are dynamic organelles that undergo fusion and fission events that ultimately control the rate of oxidative metabolism. Mitochondrial membrane proteins, such as mitofusin 1 (Mfn1), mitofusin 2 (Mfn2), and optic atrophy protein 1 (Opa1), mediate mitochondrial fusion, while phosphorylation the cytoplasmic protein dynamin-related protein 1 (Drp1) promotes its oligomerization on the mitochondrial membrane, inducing the fission of the organelle (Figure 3). While elongated mitochondria are associated to a high respiratory activity, fragmented mitochondria occur predominantly in resting cells or other situations of low energy demand [90]. 

Among the DENV NSPs, NS4B seems to be the major player in mediating the alterations in mitochondrial morphology. It inhibits the phosphorylation of Drp1, preventing its translocation to the mitochondrial membrane, ultimately inducing the accumulation of elongated mitochondria in the infected cells [73,91]. Mitochondria elongation was shown to be essential for viral replication since the induction of mitochondria fission by overexpression of Drp1 inhibited viral replication [91], while silencing Mfn2 or Drp1 expression inhibited or stimulated DENV replication, respectively [73]. In addition, DENV-infected Huh7 cells, which harbor elongated mitochondria, display increased respiratory rates associated with ATP production [91], indicating that the alterations in mitochondrial morphology induced by infection ensures the high-energy supplies required for virus replication. Then, DENV NSPs seems to promote the assembly of a complex cellular machinery next to sites of virus replication, involving close apposition of the ER with elongated mitochondria and LDs, which may facilitate virus production by providing large amounts of lipids required for viral RNA replication and virion assembly, as well as the energy supply required for virus replication.

## 6. NSP-Induced Re-Localization of Host Proteins

In addition to recruiting FASN to the RC, DENV NSPs are also involved in the re-localization of other cellular proteins. Positive- and negative-strand RNA viruses usually use nuclear components in their replication [92]. So, localization of RC in the perinuclear region seems to be important in this sense, facilitating the recruitment of nuclear proteins, which allow an efficient replication of the viral genome. A question that is raised in this context is what stabilizes RC in the perinuclear site? At least for DENV infection, this seems to be mediated by the interaction of NS4A with the vimentin scaffold [93], a component of intermediary filaments important for vesicular/organelle transport and positioning [94]. In addition to other evidence, it was shown that vimentin knockdown dissociates DENV RC, which becomes dispersed throughout the cytoplasm, suggesting its role in anchoring RC in the perinuclear region during infection [93]. 

Furthermore, it was found that the interaction of DENV NS1 with the ribosomal protein RPL-18 and its recruitment to the perinuclear region is required for viral translation and replication [95]. In another study, NS1 was also shown to promote changes in subcellular localization of GAPDH from cytoplasmic to perinuclear regions, resulting in an increase in the enzyme activity [96]. The redistribution of GADPH to sites of viral replication was also described for many other viruses, such as Japanese encephalitis virus [97], parainfluenza virus [98], hepatitis A virus [99], hepatitis C virus [100], and others [101,102]. In all cases, this re-localized pool of GAPDH was shown to bind viral RNA, which seems to be involved in the regulation of viral transcription and/or replication, and not an effect on glycolysis, which would be expected since GAPDH is a glycolysis enzyme. Further studies need to be conducted to determine if GAPDH plays a role in DENV replication, as happens with other viruses. 

With a less understood function, DENV2 NS3 subverts nuclear receptor binding protein (NRBP), a protein associated with trafficking between the endoplasmic reticulum (ER) and Golgi, recruiting it to the perinuclear region [103]. It is possible that this is important to regulate the transport of lipids and membranes to the RC, or even play a role in delivering viral particles to the Golgi/secretory pathway, but this should be investigated further.
